# Prevalence and associated factors of smoking among secondary school students in Harare Zimbabwe

**DOI:** 10.1186/1617-9625-8-12

**Published:** 2010-10-27

**Authors:** Tsitsi Bandason, Simbarashe Rusakaniko

**Affiliations:** 1 Biomedical Research and Training Institute, Harare, Zimbabwe; 2 Department of Community Medicine, College of Heath Sciences, University of Zimbabwe, Harare, Zimbabwe

## Abstract

**Background:**

There is a growing epidemic of tobacco use among adolescents in the developing world. However, there is no up to date information on smoking among adolescents. Although in the developing world concerted efforts are being made to control tobacco use, Zimbabwe does not have any documented tobacco control programmes. We estimated the prevalence of smoking among school going secondary school students in Harare, Zimbabwe.

**Methods:**

A 3-stage stratified random sampling was employed to select six participating schools and students. A descriptive analysis was conducted to describe the demographic characteristics of the participants. The prevalence of smoking was estimated and the comparison of prevalence was performed according to its associated factors. Logistic regression analysis was used to identify risk factors for smoking.

**Results:**

650 students with a mean age 16 years and 47% of them female participated. Prevalence of ever-smoked was 28.8% (95% CI 25.3 to 32.3). Prevalence of ever-smoked among males (37.8%) was significantly (p < 0.001) much higher than among females (18.5%). In the multivariate analysis, smoking was found to be statistically associated with having friends that smoke (OR 2.8), getting involved in physical fights (OR 2.3), alcohol use (OR 5.7), marijuana use (OR 8.1) and having had sexual intercourse (OR 4.4).

**Conclusions:**

The study provides recent estimates of prevalence of smoking, and indicates that there is still a high prevalence of smoking among urban secondary school students. Exposure to friends who smoke, risky behaviour like substance abuse, premarital sex and physical fights are significantly associated with smoking. Interventions to stop or reduce the habit should be implemented now and future studies should monitor and evaluate the impact of the interventions.

## Background

Adolescence is the time of life when people are more interested in taking risks and testing the boundaries of the world outside as well as their own limits. Throughout history countless adolescence has smoked tobacco [1]. This habit carries on into adult life and we find that of the 6.6 billion people on this planet, 1.3 billion are smokers and 1 billion of these are males [1]. By 2030, tobacco is expected to be the single biggest cause of death worldwide causing more deaths than HIV, malaria, tuberculosis, maternal mortality, automobile crashes, homicides and suicides combined[2]. Furthermore, it is expected that tobacco-related diseases will account for 11 percent of all deaths in developing countries by 2025[3,4]. No other consumer product has even come close to inflicting this degree of harm on the world community.

Although it has been argued that, smoking caused diseases in African countries are lower than international standards, the rise in smoking prevalence suggests a looming epidemic of smoking related diseases [5]. Information on tobacco use among young people is not available for most developing countries like Zimbabwe and the last survey was conducted ten years ago [6,7]. This means, if the effort on tobacco control is to succeed globally, progress has to be made in Zimbabwe too and the impact of intervention closely monitored and evaluated.

In this study, the aim was to estimate and characterize the prevalence of smoking among school going secondary school students in Harare, Zimbabwe, which is an urban community as well as the associated factors.

## Methods

### Setting and Target Population

The study was carried out in Harare, Zimbabwe secondary schools in February 2009. The target population was all students in Form 1 to 6 in Harare. The total population of secondary school children in Harare is estimated at 154,505 based upon the Zimbabwe 2002 Central Statistical Office Census.

### Sample Size

The minimum sample size was calculated in such a way that it accurately represented the population under survey. Based on a previous prevalence survey in Zimbabwe, the expected prevalence of smoking among adolescents was estimated to be between 18% and 31% for both males and females and it was anticipated that the non-participation rate would be less than 10%.

While it may be acknowledged that surveys based upon the study of small populations tend to generate data which does not stand the test of external validity, it should be stressed at this point that this study was not directed towards controlling for external validity. This study was directed towards producing findings which could be generalized within the context of schools in Harare, Zimbabwe. The approach also provided scope for replication of the study beyond the confines of the study's geographical location.

### Sampling Procedure

**Figure 1:**
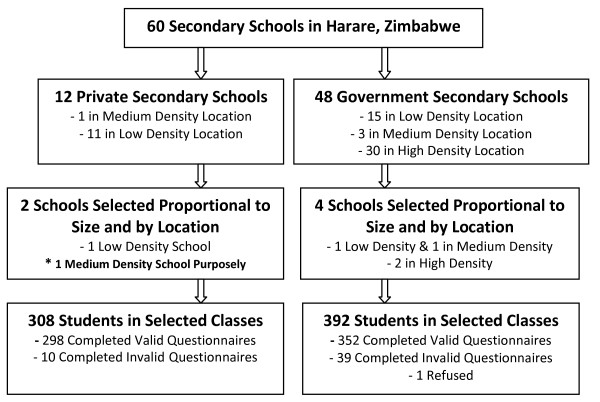
**Participant Flow Chart**.

Secondary schools registered with the Ministry of Education were stratified by type, that is, on the basis of whether they are government or private and their geographic location, that is, located in low density, medium density and high density. Six schools were selected randomly using probability proportional to size technique, where size was the number of schools by type and location (Figure 1).

A random sample of 6 schools without replacement and sampled with probabilities proportional to size was drawn using STATA 9.2 (STATA Corporation, College Station, Texas, USA).

### Participants

Each selected school was asked to provide a list of classes in each form. One class was randomly selected from each form using simple random sampling without replacement. Selected students were asked to consent to answer a questionnaire. Since the sample was constrained to include at least 120 students per school, all students in the selected classes were interviewed. If students in selected classes were absent or declined to be interviewed on the days of visit, then they were excluded from the study. Selected students were asked to consent to answer a questionnaire.

### Data collection tools and procedures

The Survey Questionnaire used in this study was adapted from the Youth Risk Behaviour Surveillance System Survey (YRBSS) questionnaire conducted by the Centers for Disease Control and Prevention. This questionnaire is almost similar to the questionnaire used for the Global Youth Tobacco Survey, but includes questions which address risky behaviour in more depth.

### Assumptions of the Study

The study was guided by the following assumptions:

i. The target population for the study had the adequate understanding and literacy to answer the questions and to provide reliable information about the problem being investigated.

ii. The sample drawn from the target population was representative for the researcher to generalize to the whole of Harare, Zimbabwe.

iii. The definition of smoking mainly used was self reported ever-smoked and this included even those who had taken one or two puffs of a cigarette.

iv. Definition of Key Terms

The following terms should be understood in the manner in which they are defined here.

**Adolescent: **A person in the state of development between puberty and maturity and being of the age 13 through to 19 years old

**Marijuana users**: These included anyone who had used cannabis, grass, hash, dope, weed, pot or a joint on any occasion

**Alcohol users: **These included anyone who had drunk any alcoholic beverage including, lager, wine, spirit or kachasu (a traditional alcoholic drink)

**Tobacco users: **These included anyone who had smoked a cigarette or used tobacco including snuff.

**Suicidal/Thoughts of Suicide: **This included anyone who has ever considered taking one's own life in the past 12 months preceding the interview.

**Physical Fights/Involvement in Physical Fighting: **This included anyone who has ever been involved in a fist fight in the past 30 days preceding the interview.

**Sports/Involvement in Sports: **This included anyone who has ever participated in any sporting team in the past 12 months preceding the interview.

**Sexual Intercourse: **These included anyone who has ever had sexual intercourse in their lifetime.

**Geographical Location: **These included residential areas with different population densities.

**Low Density Area - **These included urban area **s**uburbs with the lowest population density where people of high economic status usually reside

**Medium Density - **These included urban area **s**uburbs with medium population density where people of medium economic status usually reside

**High Density - **These included urban area **s**uburbs with the highest population density where people of the lowest economic status usually reside

Students were asked how many times they used particular substances within specified periods. This allowed for categorization of the substance use as given below. These categories were not entirely mutually exclusive but rather overlapped. This meant that a respondent who reported a substance use in the past 30 days was included in the estimates of those who had tried using the substance.

The categories used are:

**Never used **- are those who self-reported that they had never used the substance in their life time

**Ever used **- are those who self-reported that they had used the substance on one or more than one occasion

**Current users **- are those who self-reported that they had used the substance even for one day or more during the past 30 days preceding the interview.

**Peer reported users **- are those indicated by classmates that they had used the substance even in the past

### Pilot Study

The questionnaire was piloted on 20 randomly selected secondary students from an unregistered school in Harare with a similar set-up as that of registered schools. The purpose of doing this was to refine the questionnaire to suit the Zimbabwe situation thereby enhancing the construct validity. Results from this pilot generated a few modifications which included, altering the wording of the questionnaire and removal of some questions which were found to be obsolete because of cultural and structural differences with the source of the questionnaire.

### Data Analysis

Analysis was done using statistical software package called STATA 9.2 (STATA Corporation, College Station, Texas, USA). During analysis, questionnaires with contradictory or inconsistent responses were not included in the analysis of those particular questions only.

Descriptive statistics was used to describe the demographic characteristics of the respondents. The prevalence of smoking including its 95% confidence interval was calculated. Smoking prevalence was calculated separately by gender and age group. It was also demarcated into ever-smoked, current smoking and peer reported smoking and the prevalence estimated for each of these categories.

*Apriori *risk factors associated with smoking uptake included demographic characteristics (gender, race, age), socio economic status (rich, poor, place of residence), social factors (exposure to family members and friends who smoke, depression and cigarette advertising) [8]. For analysis, location of school and type of school was used to measure economic status. To assess for associated factors, comparison of prevalence was made by demographic characteristics and other social variables using Pearson's Chi-square test.

In the multivariate case, backward step-wise logistic regression was the preferred method used for selecting the risk factors. Based on literature, all the *apriori *risk factor variables were included and fitted into the model and these included:

• Demographic characteristics of respondents (gender, race, age)

• Risky Behaviour (Involvement in fights, Suicidal tendency, Sexual Intercourse)

• Other substance abuse (Alcohol use, Marijuana use)

• Economic status (as measured by geographic location of school and type of school)

• Involvement in sporting activity

• Exposure to parents or friends who smoke

The fit of the model was tested after elimination of each variable. The likelihood-ratio test was used to test the fit of the model by examining the change in the coefficient estimates due to the exclusion of the variable. Possible interaction between variables was examined and the Hosmer-Lemeshow statistic and the Pearson test was used to test if the final model was a good fit.

### Ethical Approval

Ethical approval to conduct the study was obtained from The University of Zimbabwe Joint Parirenyatwa Hospital and College of Health Sciences Research Ethics Committee, The Harare Ministry of Education Provincial Office and School Headmasters of the selected schools.

## Results

### Baseline characteristics of participants

A total of 700 students from 5 schools gave consent to participate in the study and completed the questionnaire. The total student enrolment in all the 5 schools was 5,220 implying that 13.4% of the students participated in the study. A 0.1% refusal rate was recorded and 650 (93%) of the questionnaires were valid in terms of completeness and consistency in the answers given. The baseline characteristics of the participants are shown in Table 1. The mean age of the students was 16 years and most of the students (38%) were in the 16-17 age group. 47% of the participants were female. 54% of the participants were from government schools, and 46% came from schools located in the low density area. The majority of the students (85%) were of African Origin.

**Table 1:** Summary of Participants baseline data

Characteristic	Description	Number (%)
**All participants**		650 (100%)
**Geographical Location of School**	Low density Medium density High density	299 (46.0%) 119 (18.3%) 232 (35.7%)
**School Type**	Government Private	353 (54.3%) 297 (45.7%)
**Female**		303 (46.6%)
**Age**	Mean(SD) years	15.9 (1.8)
**Age groups (years)**	13-15 16-17 18-19	215 (33.1% 245 (37.7%) 190 (29.2%)
**Race**	African Origin Caucasian Origin Asian Mixed race	555(85.4%) 51 (7.9%) 8(1.2%) 36(5.5%)
**Use Marijuana**	Yes	63(9.7%)
**Had Sexual Intercourse**	Yes	86 (13.2%)
**Drink alcohol**	Yes	407(62.6%)
**Suicidal**	Yes	95 (14.6%)
**Forms**	1 2 3 4 5 6	82 (12.6%) 127 (19.5%) 114 (17.5%) 124 (19.1%) 86 (13.2%) 117 (18.0%)

### Prevalence of Smoking

The overall prevalence of ever-smoked was 28.8% (95% CI: 25.3% to 32.3%), peer reported smoking was 26.4% (95% CI: 23.1% to 30.0%) and current-smoking was 8.5% (95% CI: 6.3 to 10.9), Table 2. Only 4.3% of all the smokers were daily smokers during the 30 days preceding the interview and they were all males, whilst 1.6% smoked at least once per week, and 23.5% smoked more seldom. This pattern suggests that most of the smokers were probably only experimenting with smoking. There was no significant difference between males and females in terms of days smoked or how the cigarettes were obtained.

**Table 2:** Prevalence of Smoking (per 100 participants), overall and according to gender

	Overall N = 650	Female n = 303	Male n = 347
**Ever Smoked**	28.8 (25.3 to 32.3)	18.5 (14.3 to 23.3)	37.8 (32.6 to 43.1)
**Current Smoker**	8.5 (6.3 to 10.9)	6.3 (3.8 to 9.6)	10.3 (7.4 to 14.1)
**Peer Reported Smokers**	26.4 (23.1 to 30.0)	12.5 (9.0 to 16.8)	38.9 (33.7 to 44.2)

### Prevalence of Smoking by Demographic Characteristics

Prevalence of ever-smoked among males was 37.8% (95%CI: 32.6% to 43.1%) and this was significantly (p < 0.001) much higher than among females which was estimated as 18.5% (95% CI: 14.3% to 23.3%). Most of the participants (49.7%) tried smoking before their 13^th ^birthday.

There was a significant difference in prevalence of ever-smoked by the type of school. In the 13-15 years age-group, 40% of the smokers were from government school as compared to 17% from private schools. However for the 16-17 years age-group, there was a reversal whereby 30% were from government and 36% from private school and in the 18-19 years age group, 30% were from government and 47% from private schools.

People of Mixed Race were found to have the highest smoking prevalence (56%), followed by those of Caucasian Origin and of African Origin (49% and 25% respectively). There was low prevalence rate among the Asians (13%).

### Prevalence of other Substance Use

Among the entire sample 62.6% (95% CI: 58.7-66.3) of the participants had drunk an alcoholic beverage, with 36.9% of these being female.

There was a small proportion of students who had used marijuana which was estimated at 9.7% (95% CI: 7.5% to 12.2%) in the entire sample.

### Prevalence of other Risky Social Behaviours

The overall prevalence of risky social behaviour like physical fighting, thoughts of suicide and sexual intercourse was generally low and was estimated as being below 15%. However, there was a significantly (p < 0.001) much higher prevalence of sexual intercourse among males which was estimated at 18.2% (95% CI: 14.2% to 22.6%) than females estimated at 7.6% (95% CI: 4.9% to 11.2%), with the overall prevalence being 13.2% (95% CI: 10.7% to 16.1%). 55.8% of the participants involved in sexual intercourse started after their 15^th ^birthday.

### Associated and Risk Factors of Smoking

The prevalence of the risky behaviour was significantly (p < 0.05) associated with smoking. Among those who reported ever smoking, 94% also drank alcohol, 29% used marijuana, 29% had sexual intercourse and 21% had been involved in physical fight. 23.5% of the smokers used alcohol more than once a week, 6.9% were involved in physical fights at least once a week, 16.1% had more than 1 sexual partner, 4.8% used marijuana more than once a week, Table 3.

**Table 3:** Prevalence of Associated Factors among Smokers

Characteristic	Never Smokers N = 463	Ever Smokers N = 187	P
**Drink Alcohol**			
No	232 (50.1%)	11 (5.9%)	**< 0.001**
Yes	231 (49.9%)	176 (94.1%)	
**Ever use marijuana**			
No	454 (98.1%)	133 (71.1%)	**< 0.001**
Yes	9 (1.9%)	54 (28.9%)	
**Physical fights**			
No	429 (92.7%)	148 (79.1%)	**< 0.001**
Yes	34 (7.3%)	39 (20.9%)	
**Sports**			
No	137 (29.6%)	33 (17.7%)	**0.002**
Yes	326 (70.4%)	154 (82.3%)	
**Sexual Intercourse**			
No	431 (93.1%)	133 (71.1%)	**< 0.001**
Yes	32 (6.9%)	54 (28.9%)	
**School Location**			
Low Density	193 (41.6%)	106 (56.7%)	**< 0.001**
Medium Density	63 (13.6%)	56 (29.9%)	
High Density	207 (44.7%)	25 (13.4%)	
**School Type**			
Government	303 (65.4%)	50 (26.7%)	**< 0.001**
Private	160 (34.6%)	137 (73.3%)	
**Sex**			
Female	247 (53.3%)	56 (29.9%)	**< 0.001**
Male	216 (46.7%)	131 (70.1%)	
**Race**			
African Origin	414 (89.4%)	141 (75.4%)	
Caucasian Origin	26 (5.6%)	25 (13.4%)	**< 0.001**
Asian	7 (1.5%)	1 (0.5%)	
Mixed race	16 (3.5%)	20 (10.7%)	
**Suicidal**			
No	407 (87.9%)	148 (79.1%)	**0.004**
Yes	56 (12.1%)	39 (20.9%)	
**Friends who smoke**			
No	342 (73.9%)	46 (24.6%)	**< 0.001**
Yes	121 (26.1%)	141 (75.4%)	
**Parents who smoke**			
No	354 (76.5%)	122 (65.2%)	**0.003**
Yes	109 (23.5%)	65 (34.8%)	

On univariate analysis, one was more likely to be a smoker if they were exposed to friends who smoke (OR 8.7, 95% CI: 5.9-12.8) and had parents who smoke (OR 1.7, 95% CI: 1.2-2.5), Table 4. High socio-economic status as measured by being from a private school gave an OR of 5.2 (95% CI: 3.6-7.6) and was a significant risk factor and had a population attributable fraction (PAF) of 89.2%.

**Table 4:** Odds Ratio of Risk Factors for Smoking Among Adolescents in Harare, Zimbabwe in 2009

Factors	Univariate Analysis			Multivariate Analysis		
	
	Odds Ratio	95%CI	P	Odds Ratio	95%CI	P
**Sex**						
Male	2.68	1.86-3.84	**< 0.001**	-	-	-
**Age (years)**						
13-15	1					
16-17	1.37	0.88-2.12	0.154	-	-	-
18-19	2.76	1.78-4.29	**< 0.001**			
**Race**						
African Origin	1					
Caucasian Origin	2.82	1.58-5.05	**< 0.001**	-	-	-
Asian	0.42	0.05-3.43	0.418			
Mixed race	3.67	1.85-7.28	**< 0.001**			
**Type of School**						
Government	1					
Private	5.19	3.56-7.56	**< 0.001**	-	-	-
**Geographical Location of School**						
Low density	1					
Medium density	1.61	1.05-2.49	0.029			
High Density	0.22	0.14-0.35	**< 0.001**	0.27	0.15-0.52	**< 0.001**
**Friends who smoke**						
Yes	8.66	5.85-12.82	**< 0.001**	2.75	1.73-4.36	**< 0.001**
**Parents who smoke**						
Yes	1.73	1.20-2.50	0.004	-	-	-
**Use Marijuana**						
Yes	20.48	9.85-42.57	**< 0.001**	8.08	3.46-18.83	**< 0.001**
**Involved in Physical fight**						
Yes	3.32	2.02-5.46	**< 0.001**	2.29	1.24-4.24	0.008
**Drink Alcohol**						
Yes	16.06	8.51-30.34	**< 0.001**	5.72	2.85-11.46	**< 0.001**
**Had Sexual Intercourse**						
Yes	5.46	3.39-8.82	**< 0.001**	4.43	2.25-8.74	**< 0.001**
**Suicidal**						
Yes	1.92	1.22-3.00	0.005			

Demographic risk factors for being a smoker were, being male (OR 2.7, 95% CI: 1.9-3.8), being over 17 years (OR 2.8, 95% CI: 1.8-4.3), and being of non-African Origin (OR 3.7, 95% CI: 1.9-7.3).

In the multivariate analysis, having friends who smoke (OR 2.8, 95% CI: 1.7-4.4), being involved in physical fights gave (OR 2.2, 95% CI: 1.2-4.2), using marijuana (OR 8.1, 95% CI: 3.5-18.8), drinking alcohol (OR 5.7, 95% CI: 2.9-11.5) and being involved in sexual intercourse (OR 4.4, 95% CI: 2.2-8.7) all remained as a significant risk factors for smoking, whilst coming from a school located in the high density remained significantly protective (OR 0.3, 95% CI: 0.1-0.5), Table 4.

Controlling for school type, this association remained significant except for physical fights in government schools.

Separate models were fitted for government and private schools to cater for the different socio-economic status of the participants and the difference in the significant risk factors.

## Discussion

This study showed that there is substantial burden of experimental smoking among secondary school children in Harare, Zimbabwe. In an average class of 40 pupils (which is the standard class size in Zimbabwe) we would expect that at least 25% of them have experimented with smoking and 7% are daily smokers. The prevalence of ever-smoked is above that reported in the previous survey carried out in 1999 Global Youth Tobacco Survey Zimbabwe, and this is a cause for concern [9]. It is well known that experimentation with danger is crucial to the adolescent experience and they start this as an act of rebellion or as a sign of maturity but it ends up being an addictive behaviour [10]. It is still a fact that adolescents need to open their eyes and ears to what is really going on their lives if they indulge in this risky behaviour [2]. This may mean using education from an early age to keep them safe rather than deny or ignore the problem. The different risks and associated factors are discussed below.

There was increased prevalence of smoking of 38% among males and 19% among females as compared to the last reported prevalence of 31% among male students and 18% among female students living in an urban area [6]. Consistent with other studies, the prevalence of smoking was higher among males and the gap between males and females seemed not to be narrowing as previous studies intimated [11]. This difference in prevalence between genders might be due to social and cultural acceptance of smoking among males rather than females in Zimbabwe. The potential long-term health consequences are not even considered when the boys want to satisfy their need for social interaction [8,12].

Although international trends indicate that prevalence of smoking is higher among the non-Caucasian Origin groups [6], this study showed that the prevalence is higher among the non-African Origin groups which concurs with a study carried out in South Africa [13,14]. The low prevalence rate among the African Origin group could be explained by the economic situation in Zimbabwe which has left students without any disposable cash, especially those from high density areas. Ethnic differences in smoking prevalence and initiation might be due to differences of social factors [12,13]. This might have been further exacerbated by the issue of acculturation, whereby pupils of African Origin in former schools of pupils of Caucasian Origin adopted the cultural characteristics of the non-African Origin ethnic groups as also exhibited in the study among Chinese American minors [12].

The study showed that some 22.5% students started smoking before they were 10 years which is the same trend seen internationally and in the previous study carried out in Zimbabwe,[9] where one-third of the smokers started smoking whilst they were still in their teens. The issue of increase in the prevalence of smoking across age groups might be explained by the addictive nature of the habit and therefore students fail to stop and experience withdrawal symptoms during times of abstinence, therefore they continue smoking up to adulthood [15]. It therefore follows that a programme that successfully reduces youth smoking is likely to yield a good long-term public health benefit as most of these people who become smokers in adulthood start whilst they are still in their youth [16].

Most adolescents have the sense and self-preservation to avoid indulging in any risky behaviour, because once one indulges in experimental smoking, they are most likely to indulge in experimental drinking alcohol, using marijuana, sexual intercourse and physical fights [2,8,17]. In this study this association was found to be quite strong.

On sports, this study did not find the expected inverse association between sports and smoking [18]. This suggests that sports are not perceived as an important motivating factor of the need to fulfill social intimacy or interaction [8].

Although there were several social risk factors for smoking, high among them were exposure to friends who smoke. Several studies have concluded that adolescents who have friends who smoke are most likely to be smokers too, and the same was confirmed in this study too [13,19]. Also, having a parent who is a smoker came out as being a significant independent risk factor for smoking. The same finding was obtained among adolescents in United States, where having father or mother who is a smoker was found to significantly increase the risk [20]. This effect of social risk factors may be due to the fact that during adolescence, children are more readily likely to succumb to peer social influences to satisfy their need for social interaction with their peer group [8]. Therefore, any interventional strategy developed should take the effect of peer pressure into consideration.

Smoking and alcohol use are rarely problematic for children with positive psychological

wellbeing [8,18]. All available research suggests that the majority of children who develop serious addictive problems are those who are depressed or those given too much freedom. As found in this study, those in private schools might have been exposed to a lot of freedom to the extent of being spoilt and searched for another high than those with limited resources [19]. Whilst some depressed children used smoking and drinking as a way to ease difficulties in social situations. A similar finding was obtained among Chinese adolescents, where depression was found to be significantly associated with smoking for both boys and girls [21].

The study concurred with other studies in that, the male gender was more at risk. However, in the Zimbabwe context, smoking is more acceptable among males than females because of cultural and social norms. Therefore, girls are most likely to under-report or not indulge in this social undesirable behaviour [17].

Alcohol was the most widely used of the substances investigated as compared to marijuana. Use of alcohol increased as the students got older.

Although there is paucity of smoking prevalence data in Zimbabwe and other developing countries, in general, the findings from this study indicate that the risk factors as seen in Western countries are similar to those found in this study [10,13]. Although they might have been other cultural factors which were not investigated in this study which might affect the relationship with smoking, the findings of this study suggest that intervention strategies designed for Western countries can also be effective in the Zimbabwe situation. This includes having a school environment that is positive, that is, an environment that stimulates rather than depress the students [8,16,19].

This study may have over-estimated the prevalence by using self-reports, however, most researchers concur that self-reports are accurate estimates of experimental smoking which can later lead to addicted smokers [22]. The prevalence of current smokers seemed to be lower than expected, however, this was expected, since the period evaluated was a school term period, and the students might have had no access to cigarettes as most of them bought from a shop, which they could not do whilst in uniform.

Although the cross-sectional design of the study provided evidence that suggested a relationship between smoking and the study factors, it gave limited inference of the causal relationship with these factors. Therefore it was not possible to determine whether the exposure preceded or followed the initiation of smoking. This also limited the study's need to distinguish between normal experimentation and avoidance of behaviour that needs help. This information could only have been obtained through qualitative study, which could have given an insight on why adolescents need to take the risk, and helped understand whether the adolescents do know the dangers or they know and do not care [23]. However, since the study was self-sponsored, this limited any further work to be carried out.

Selection bias might also have occurred through the selection of a sample that was not entirely representative of the population under study. It was not possible to cover all levels of school locations and there was no equal distribution of schools by location for both government and private schools.

## Conclusion

Apart from providing recent estimates on the prevalence of smoking, use of illicit substances and involvement in risky behavior, this study indicates that, there is still a high prevalence of smoking among adolescents in Zimbabwe. The odds that one is a smoker are increased when students have friends and parents who smoke. Also, there is a significant association between smoking and other risky behaviour like alcohol use, marijuana use and premarital sex.

Although assisting young people to avoid smoking is a widely endorsed goal of public health already, no adequate action has been taken to develop interventions that stop or reduce this habit and to make informed decisions in Zimbabwe. Since the findings are almost similar to those found in Western countries, high cigarette prices and laws against youth access or Adolescent Tobacco Education can be recommended as interventional strategies which work [38]. Furthermore, if health policy makers need to reduce the impact of tobacco related diseases like Tuberculosis, strategies for controlling tobacco use should be implemented now. Future studies should be implemented to monitor and evaluate the impact of the interventions.

## Competing interests

The authors declare that they have no competing interests.

## Authors' contributions

TB contributed to the design of this study, conducted an exploratory survey of the literature, recruited participants, conducted the survey and analysis and drafted this paper. SR supervised the overall project. All authors read and approved the final manuscript.
